# Cancer Vaccine Strategies in Non-Small Cell Lung Cancer

**DOI:** 10.3390/vaccines14070562

**Published:** 2026-06-25

**Authors:** Rogelio N. Velasco, Pragadeesh Thamaraiselvan, Edoardo Garbo, Silvia Novello, Francesco Passiglia

**Affiliations:** 1 Thoracic Oncology Department, Lung Center of the Philippines, Metro Manila 1101, Philippines; rogervelascojr@gmail.com; 2 Department of Medical Oncology, Cancer Institute (WIA), Chennai 600032, India; pragadeesh.t12@gmail.com; 3 Department of Oncology, University of Turin, San Luigi Gonzaga University Hospital, 10043 Orbassano, Italysilvia.novello@unito.it (S.N.)

**Keywords:** cancer vaccines, immunotherapy, lung cancer, non-small-cell lung cancer, vaccines, immunology

## Abstract

Despite significant improvement in long-term survival with the advent of immunotherapy, a substantial proportion of lung cancer patients develop primary and acquired resistance. Among emerging strategies to overcome this challenge, cancer vaccines represent a promising approach, especially for non-small-cell lung cancer (NSCLC). A variety of vaccine platforms have been investigated, including nucleic acid-based vaccines, peptide vaccines, dendritic cell vaccines, and viral vector-based approaches. To date, cancer vaccines have not demonstrated consistent survival benefit in large randomized trials, and their clinical application remains limited. Challenges include high production costs, complexity in manufacturing, and issues related to drug stability and scalability. However, several ongoing early-phase trials show promising signals for several platforms, as new tools and technology become available to optimize neoantigen selection, vaccine production, efficacy, and safety. In this review, we summarize the current evidence of vaccines in NSCLC treatment across different stages and therapeutic settings.

## 1. Introduction

Lung cancer is the leading cause of cancer and cancer-related death worldwide, with an estimated 2.5 million new cases and 1.8 million deaths reported in 2022 [1]. Despite significant advancements in treatment over the last few decades, particularly with increasing use of targeted therapies and immunotherapy, overall outcomes remain suboptimal [2]. The 5-year survival of patients with lung cancer continues to be less than 30%, as reported in the SEER database [3]. Therefore, there is an ongoing need to develop novel therapeutic strategies to improve patients’ survival.

Immunotherapy aims to harness the host immune system to recognize and eliminate tumor cells. In lung cancer, immune checkpoint inhibitors (ICIs) have become a cornerstone of treatment for patients without an actionable driver mutation, across both locally advanced and metastatic settings [4,5,6]. However, a significant proportion of patients do not derive a durable benefit, highlighting the need for alternative or complementary immunotherapeutic approaches [7,8].

Cancer vaccines represent one such strategy, designed to stimulate an anti-tumor immune response by presenting tumor-associated or tumor-specific antigens to the cells of the immune system. In oncology, vaccines may be preventive, such as the HPV vaccines, or therapeutic, like Sipuleucel T-cell for prostate cancer [9,10]. In non-small-cell lung cancer (NSCLC), the use of therapeutic cancer vaccines remains an area of active investigation, with multiple strategies currently under evaluation, and in this narrative review, we aim to gather the latest evidence in this expanding field of research.

The major completed clinical trials evaluating therapeutic vaccines in NSCLC across different settings have been summarized in Table 1, Table 2 and Table 3, while the ongoing trials have been listed in Table 4.

## 2. Biology and Scientific Rationale of Lung Cancer Vaccines

Cancer vaccines represent a promising approach that harnesses the immune system to selectively recognize and target cancer cells. Therapeutic cancer vaccines can be designed to be recognized as *tumor-associated antigens* (overexpressed in cancer cells) or as *tumor-specific antigens* (arising from genetic alterations unique to the cancer) [11].

Cancer vaccines enhance antitumor immunity by restoring and amplifying the cancer–immunity cycle, beginning with improved antigen presentation, followed by T-cell recruitment and effective tumor cell killing. Vaccine-derived or tumor-released antigens are taken up by dendritic cells (DCs), processed, and then presented on MHC class I and II molecules to enable the priming of CD8^+^ and CD4^+^ T-cells, respectively [12,13]. Adjuvants play a critical role by activating DCs, increasing co-stimulatory molecule expression, and promoting proinflammatory cytokine production, thereby ensuring the delivery of the three essential signals required for optimal T-cell activation [12,14]. Once primed, effector T-cells expand and traffic to the tumor microenvironment, guided by chemokines and inflammatory cues that vaccines can enhance. Within the tumor, CD8^+^ cytotoxic T-cells recognize antigen–MHC complexes on cancer cells and mediate direct killing, while CD4^+^ T-cells support effector function and recruitment. Importantly, this process can be self-amplifying, as tumor cell death releases additional antigens that further propagate immune activation [12,15]. However, tumors often evade this cycle through impaired antigen presentation, deficient T-cell priming, or restricted trafficking; cancer vaccines are designed to overcome these barriers and convert immunologically “cold” tumors into “hot” T-cell-inflamed states [12].

Multiple vaccine platforms have been explored in NSCLC, encompassing both shared antigen-based approaches targeting tumor-associated drivers and personalized strategies tailored to the unique molecular profile of an individual patient’s tumor. The latter relies heavily on next-generation sequencing technologies to identify patient-specific neoantigens and inform vaccine design. Nucleic acid-based vaccines, including DNA and RNA vaccines, are widely studied due to their stability and adaptability, which use the host’s own protein synthesis machinery [16,17]. DNA vaccines can induce antigen-specific immune responses and are relatively straightforward to produce, whereas RNA vaccines can generate a more potent immune response compared with DNA vaccines, but are limited by their inherent instability. While mRNA vaccines only need to cross the cell membrane, DNA vaccines must traverse the cell membrane of the antigen-presenting cells (APCs), such as dendritic cells, and migrate to the nucleus for transcription and be exported to the cytoplasm for translation. In addition, nucleic acid vaccines are easier to manufacture, conferring less time and resources in production compared to traditional protein- or peptide-based vaccines [18]. Dendritic cell vaccines are produced by harvesting precursor cells, which undergo maturation and through which antigens are introduced and subsequently injected into the patient [19]. Peptide-based vaccines offer high specificity and ease of manufacturing, although they may exhibit reduced immunogenicity. Tumor cell-based vaccines present a broad array of antigens, potentially enhancing immune recognition; however, their production is technically challenging. In situ cancer vaccines, on the other hand, stimulate immune responses through the release of tumor antigens from dying or dead tumor cells following local tumor destruction [20].

## 3. Vaccines in Early-Stage and Locally Advanced NSCLC: An Opportunity to Prevent or Delay Recurrent Disease

The current standard of care for patients with *EGFR*/*ALK wild-type*, locally advanced and resectable NSCLC includes surgical resection with mediastinal nodal dissection, along with perioperative or adjuvant chemoimmunotherapy [6]. For patients with locally advanced unresectable *EGFR*/*ALK wild-type* disease, the treatment of choice is concurrent chemoradiotherapy, followed by one year of maintenance ICI immunotherapy with durvalumab [21,22]. Despite these multimodal approaches, a significant proportion of patients with stage III disease eventually relapse after definitive treatment. Several vaccine-based strategies have been evaluated in the adjuvant setting to reduce the risk of recurrence following curative-intent therapy. **Tecemotide** (BLP25), a liposome-based peptide vaccine targeting MUC1 (Mucin-1, a tumor-associated antigen), is one of the most extensively studied vaccines in this setting [23]. In a phase III randomized trial, tecemotide was compared with placebo as maintenance therapy in patients with at least stable disease following chemoradiotherapy. The study did not meet its primary endpoint of overall survival (median OS 25.6 vs. 22.3 months; HR 0.88, 95% CI 0.75–1.03, *p* = 0.123). A subgroup analysis suggested a survival benefit of the vaccine among patients who received concurrent chemoradiotherapy, as opposed to sequential chemoradiation [24]. However, this was not confirmed in subsequent studies, including a phase I/II trial in the Japanese population [25].

In the post-surgical setting, the **MAGE-A3** cancer vaccine, a recombinant protein vaccine targeting the tumor-associated antigen MAGE-A3, combined with the immunostimulant AS15, was evaluated in 2312 patients with resected stage IB-III MAGE-A3-positive NSCLC. In this phase III MAGRIT trial, patients were randomized to receive the vaccine or a placebo. The study did not demonstrate an improvement in the disease-free survival (median DFS 60.5 vs. 57.9 months; HR 1.02, 95% CI 0.89–1.18, *p* = 0.74) [26]. It is important to note that these studies were conducted prior to the routine use of adjuvant immunotherapy, and the role of vaccines in combination with maintenance immunotherapy needs to be defined.

The safety and immunogenicity of a neoantigen-presenting autologous dendritic cell vaccine have also been investigated in patients with resected NSCLC. In a small study, antigen-specific T-cell responses, assessed by interferon-gamma secretion, were observed in five of six evaluable patients, with no significant toxicity reported [27]. DPV-001 (DRibble vaccine), composed of tumor-derived autophagosomes containing multiple tumor antigens, has been evaluated in patients with stage III NSCLC following definitive therapy. The vaccine was well tolerated and demonstrated robust immunogenicity, although clinical efficacy data are not yet available [28,29].

Ongoing studies are further evaluating vaccine strategies in the adjuvant setting, including trials investigating personalized and multi-agent approaches. In the MIDRIXNEO-LUNG study, an autologous dendritic cell vaccine encoding patient-specific neoantigens demonstrated acceptable safety and induced neoantigen-specific T-cell responses in resected NSCLC patients. However, the full results of the study are not yet available [30]. Also, the ongoing phase III INTerpath-002 trial is evaluating the combination of the individualized mRNA neoantigen vaccine V940 (mRNA-4157) with pembrolizumab in resected stage II-IIIB NSCLC, following surgery and adjuvant chemotherapy [31]. Overall, vaccine strategies in the non-metastatic setting have shown immunogenicity, but have not yet translated into a consistent survival benefit.

## 4. Metastatic NSCLC: The Use of Vaccines Across the Treatment Continuum

Immunotherapy-based treatment remains the standard-of-care for patients without actionable genomic alterations (AGAs) with metastatic NSCLC. However, primary or acquired resistance inevitably develops among the majority of patients due to several mechanisms. Primary resistance may be from a low tumor antigen load, impaired antigen presentation, aberrant T-cell activation, and the presence of an immunosuppressive tumor microenvironment. Acquired resistance mechanisms may occur after an initial response, and may be due to the exhaustion of T-cell function, reduction in T-cell numbers, and loss of neoantigens during treatment [32]. Thus, novel treatment strategies are needed to overcome these challenges in the metastatic setting, in addition to standard-of-care.

In other NSCLC subtypes harboring AGAs, vaccine strategies remain largely in early development. Alterations such as *EGFR* and *ALK* represent promising targets for antigen-specific or personalized vaccine platforms, with the potential to expand therapeutic options beyond targeted therapies and to restore antitumor immunity in these typically ‘immune-cold’ tumors [33,34].

## 5. Therapeutic Vaccines as a Maintenance Strategy

Several vaccine strategies have been evaluated as maintenance therapy, following initial chemotherapy in patients with advanced NSCLC. **Belagenpumatucel-L**, an allogenic whole-tumor-cell vaccine with a TGF-β2 antisense construct, was evaluated in a large phase III randomized trial in 532 patients with advanced NSCLC following platinum-based chemotherapy. The study did not show an improvement in overall survival compared with placebo (median OS 20.3 vs. 17.8 months; HR 0.94; *p* = 0.594) [35]. **CIMAvax-EGF**, a recombinant human epidermal growth factor conjugate vaccine designed to induce anti-EGF antibodies, was evaluated as switch maintenance therapy in a phase III randomized trial in 405 patients with advanced NSCLC. The study did not demonstrate a statistically significant improvement in overall survival in the safety population, compared with best supportive care (median OS 10.8 vs. 8.9 months; HR 0.82, 95% CI 0.66–1.03; *p* = 0.10), although the vaccine was generally well tolerated [36].

**Racotumumab**, an anti-idiotype vaccine targeting the NeuGcGM3 tumor-associated ganglioside, was evaluated in 176 patients with advanced NSCLC who had stable disease after first-line chemotherapy. In a randomized phase II/III study, racotumumab demonstrated a modest improvement in progression-free survival (5.3 vs. 3.9 months; HR 0.73; *p* = 0.039) and overall survival (8.2 vs. 6.8 months; HR 0.63; *p* = 0.004) compared with placebo [37]. A subsequent phase III RANIDO trial, which was designed as a non-inferiority study comparing racotumumab and nimotuzumab with docetaxel, the results did not demonstrate a clear clinical advantage, though the non-inferiority cut-off was met. The choice of comparator with docetaxel, which is not typically used in the maintenance setting, limits the interpretability of these findings in the context of maintenance therapy [38].

**Vx-001**, a peptide vaccine targeting the universal tumor antigen Telomerase Reverse Transcriptase (TERT), was investigated in a phase II randomized trial in 221 patients with advanced NSCLC expressing hTERT, who did not progress after first-line platinum-based doublet chemotherapy. The study did not meet its primary endpoint of overall survival (median OS 14.3 vs. 11.3 months; HR 0.97, 95% CI 0.70–1.34), and the time to treatment failure was similar between the vaccine and placebo arms, despite a favorable safety profile [39]. Other smaller studies have explored maintenance vaccine strategies with limited clinical impact. Early-phase studies of second-generation hTERT-based vaccines such as UV1 have shown acceptable safety and encouraging survival signals in small cohorts [40], but these findings require validation in larger randomized trials.

## 6. Therapeutic Vaccine-Based Upfront Combinations

In addition to maintenance strategies, several vaccines have been evaluated in combination with chemotherapy, and more recently, with immune checkpoint inhibitors, in the first-line metastatic setting. **TG4010**, a modified vaccinia Ankara viral vector vaccine encoding MUC1 and interleukin-2, is one of the most extensively studied vaccines in this setting. In the phase IIb/III TIME trial, the addition of TG4010 to platinum-based chemotherapy, compared with placebo in 222 patients, resulted in modest improvements in progression-free survival (5.9 vs. 5.1 months; HR 0.74, 95% CI 0.55–0.98; *p* = 0.019), with no statistically significant improvement in overall survival, though it was not powered for this secondary endpoint. The vaccine was generally well tolerated [41]. Subsequent phase II studies have evaluated TG4010 in combination with chemotherapy and Nivolumab in 44 advanced NSCLC patients, demonstrating manageable safety and modest clinical activity, supporting further evaluation of this approach [42].

**DCVAC/LuCa**, an autologous dendritic cell-based vaccine pulsed with tumor antigens, has also been evaluated in combination with immune stimulators (Interferon-α and Hydroxychloroquine) and chemotherapy in a randomized phase II trial. The addition of the DCVAC/LuCa showed an improvement in overall survival compared to standard-of-care chemotherapy (median OS 15.5 vs. 11.8 months; HR 0.55, 95% CI 0.33–0.93), with minimal additional toxicity [43]. While these findings are encouraging, they require confirmation in larger randomized studies. Personalized neoantigen vaccines, such as **NEO-PV-01,** have been evaluated in combination with chemotherapy and pembrolizumab in early-phase studies and have shown acceptable safety, with encouraging response rates [44]. Similarly, PDC*lung01, an allogenic plasmacytoid dendritic cell-based vaccine, has been evaluated in patients with resected and metastatic NSCLC in combination with anti-PD1 therapy. In the advanced disease cohort, the vaccine demonstrated an objective response rate of 51% and a median progression-free survival of 9 months, with an acceptable safety profile, suggesting potential activity for this approach in combination with immunotherapy [45].

Ongoing studies are evaluating newer vaccine platforms in combination with first-line therapies. These include DNA-based vaccines such as STEMVAC, autologous dendritic cell vaccines such as PEP-DC, and personalized multi-epitope peptide vaccines such as Microlyvaq, which are being investigated with immune checkpoint inhibitors and chemotherapy in patients with advanced NSCLC (NCT05242965, NCT05195619, NCT07285434).

## 7. Therapeutic Vaccine-Based Combinations for Subsequent Lines

The role of cancer vaccines has also been explored in patients with NSCLC who develop resistance to immune checkpoint inhibitors, with the aim of overcoming immune escape and restoring anti-tumor T-cell responses. **OSE2101**, a multi-epitope peptide-based vaccine targeting multiple tumor-associated antigens (Her2/neu, CAE, MAGE-A2, MAGE-A3, and p53), was evaluated in the phase III ATALANTE-1 trial in 219 patients with HLA-A2-positive, *EGFR/ALK*-negative advanced NSCLC, who had progressed after prior immunotherapy (with or without chemotherapy). Patients were randomized to receive OSE2101 or standard-of-care chemotherapy. The study did not demonstrate a significant improvement in overall survival (median OS 8.8 vs. 8.3 months; HR 0.86, 95% CI 0.62–1.19; *p* = 0.36), although the vaccine showed a good safety profile [46].

**UCPVax**, a peptide vaccine designed to induce CD4+ T-helper-1 responses against TERT, has been evaluated in patients with advanced NSCLC, following progression on chemo-immunotherapy. In a randomized phase II trial comparing UCPVax combined with nivolumab versus chemotherapy, the study was prematurely terminated due to a lack of efficacy. The vaccine arm demonstrated inferior outcomes, with lower response rates, shorter progression-free survival, and reduced overall survival, indicating limited activity in this setting [47]. **TEIPP24** is a peptide vaccine against T-cell epitopes associated with impaired peptide processing (LRPAP1). In a phase I/II study involving patients with HLA-A*02:01-positive NSCLC who progressed after immune checkpoint blockade, TEIPP24 demonstrated the induction of an immune response in a majority of the patients. However, clinical activity was modest, with low objective response rates and a median overall survival of 9.4 months [48].

Other approaches targeting immune resistance are currently under investigation, including vaccines directed against shared oncogenic drivers such as KRAS, and personalized neoantigen-based strategies combined with immune checkpoint blockade in early-phase studies (NCT06015724). Overall, vaccine strategies in the IO-resistant setting have not yet demonstrated meaningful clinical benefit, although novel approaches targeting immune escape mechanisms are an area of active investigation.

Cancer vaccines have also been evaluated in patients with advanced NSCLC, who have progressed after multiple lines of therapy, including chemotherapy and immune checkpoint inhibitors, where treatment options are limited. **Viagenpumatucel-L** (HS-110), an allogenic tumor cell-based vaccine, has been investigated in combination with immune checkpoint inhibitors in previously treated NSCLC. In early-phase trials, responses were observed in a subset of patients, particularly among immune responders, with an acceptable safety profile [49,50].

**NEO-PV-01**, a personalized neoantigen vaccine, has been investigated in combination with nivolumab in patients with advanced solid tumors, including NSCLC. In early-phase studies, the vaccine demonstrated immunogenicity, and in the NSCLC cohort of 18 patients, showed encouraging response rates (Objective Response Rate, ORR 39%), with a median PFS of 8.5 months, although these findings reflect results from a small, non-randomized study [51]. **UCPVax** has also been evaluated in heavily pretreated patients with NSCLC. In a phase I/II study, the vaccine demonstrated a favorable safety profile, with a median overall survival of 9.7 months and a disease control rate of 39%, although the ORR was low (1.9%) [52]. **Neo-DCVac**, a neoantigen peptide-pulsed autologous dendritic cell vaccine, has been evaluated in a small phase I study in patients with advanced NSCLC. The vaccine demonstrated acceptable safety, with preliminary signals of clinical activity (ORR 25%, median PFS 5.5 months, and median OS 7.9 months), although these findings require validation in larger studies [53].

Emerging vaccine platforms are also being evaluated in early-phase studies, with published results not yet available. These include the mRNA-based vaccines, such as BI 1361849, as well as other next-generation approaches combining vaccines with immune checkpoint inhibitors in previously treated NSCLC. While several vaccine strategies in the refractory setting have shown promising immunogenicity and occasional clinical responses, these findings remain exploratory, and none have shown a definitive survival benefit. Notably, there are no large randomized phase III trials evaluating cancer vaccines exclusively in the refractory setting, and most available evidence is limited to early-phase studies with small sample sizes.

## 8. Discussion

Despite promising immunological activity observed across several vaccine platforms, clinical outcomes in NSCLC have been largely disappointing, with most randomized studies failing to demonstrate a significant benefit. Nevertheless, the field has continued to evolve, with newer platforms seeking to overcome the limitations of earlier vaccine strategies. Earlier approaches largely focused on shared tumor-associated antigens and generally failed to demonstrate meaningful clinical efficacy, likely due to their limited immunogenicity. In contrast, newer platforms, such as personalized neoantigen vaccines, nucleic acid-based approaches, and dendritic cell-based strategies, are showing the ability to generate more robust and specific immune responses.

The widespread adoption of ICIs has also reshaped lung cancer’s therapeutic landscape, positioning vaccines more as potential immune enhancers that can synergize with checkpoint blockade. Patient selection is likely to be important for the future development of vaccines in NSCLC. To date, there is limited evidence that histological subtype alone predicts vaccine efficacy. Tumors with a higher mutation burden and greater neoantigen diversity may be more suitable for vaccine-based approaches, whereas some vaccine platforms are further limited by HLA restrictions that reduce their applicability across a broader patient population. At present, no validated biomarker exists to guide the selection of a specific vaccine platform in NSCLC, although factors such as antigen expression (e.g., MUC1, MAGE-A3), HLA status, and oncogenic driver alterations have been used for patient selection in individual studies. In this context, personalized neoantigen vaccines are especially attractive, as they may improve immunogenicity, exploit tumor heterogeneity, and expand T-cell repertoires, particularly when combined with ICIs.

Novel preventive strategies are also emerging, including preventive approaches aimed at reducing the risk of second primary lung cancers or recurrence following very early-stage disease, such as the LungVax vaccine currently under investigation [54]. In parallel, combinatorial strategies integrating cancer vaccines with adoptive T-cell therapies are being explored to further enhance antitumor immunity.

Several factors may explain the limited success of vaccines in advanced disease, including the presence of an immunosuppressive tumor microenvironment, T-cell exhaustion, and prior treatment-related immune dysfunction in heavily pretreated patients. These challenges have shifted attention toward earlier-stage disease, where tumor burden is lower and immune competence is relatively preserved, potentially increasing the likelihood of clinical benefit. However, important limitations remain, including HLA restriction for many vaccine platforms, which may limit broad applicability [55], as well as significant challenges related to manufacturing complexity, turnaround time, cost, and accessibility—particularly for personalized approaches. The potential role of vaccines in immunologically “cold” tumors, such as *EGFR*/*ALK*-altered NSCLC, remains an area of active investigation, as these strategies could potentially help overcome intrinsic resistance to immunotherapy by enhancing antigenicity and T-cell infiltration.

**Table 1:** Completed clinical trials on peptide-based vaccines.

NCT Number	Population	Intervention	Primary Outcome	Phase and Sample Size	Results
**A. Anti-Mucin vaccine—Tecemotide**					
NCT00409188 [24]	Inoperable stage III NSCLC after chemoradiotherapy with at least stable disease	Tecemotide (L-BLP25 Liposome Vaccine) versus placebo (2:1) (liposome-based peptide vaccine against MUC1)	OS	Phase III N = 1239 (829 in vaccine arm)	Vaccine vs. placebo Grade 3+ AE—33% vs. 36% Median OS—25.6 vs. 22.3 months (HR 0.88, 95% CI 0.75–1.03, *p* = 0.123)
NCT01015443 [56,57]	Inoperable stage III NSCLC after chemoradiotherapy with at least stable disease in the East Asian population	Tecemotide (L-BLP25 Liposome Vaccine) versus placebo (2:1) (liposome-based peptide vaccine against MUC1)	OS	Phase III N = 285 (191 in vaccine arm)	Vaccine versus placebo Serious TEAE—17.8% vs. 22.3% Median PFS—7.0 vs. 8.7 months Median OS—not reached in both arms (HR—1.03, 95% CI 0.55–1.93, *p* = 0.921)
NCT00960115 [25]	Inoperable stage III NSCLC after chemoradiotherapy with at least stable disease	Tecemotide (L-BLP25 Liposome Vaccine) versus placebo (2:1) (liposome-based peptide vaccine against MUC1)	OS	Phase I/II Randomized N = 172 (114 in vaccine arm)	Vaccine vs. Placebo Grade 3+ AE—25.4% vs. 17.5% Median PFS—11.6 vs. 8.0 months (HR 0.95, 95% CI 0.66–1.37) Median OS—32.4 vs. 32.2 months (HR 0.95, 95% CI 0.61–1.48, *p* = 0.83)
NCT00828009 [23]	Inoperable stage IIIA or IIIB NSCLC after chemotherapy and radiation therapy	BLP25 Liposome Vaccine (Tecemotide) and Bevacizumab (liposome-based peptide vaccine against MUC1)	Safety and tolerability	Phase 2 N = 33	Grade-4 AE—3.0% SAE—33.3% Median PFS—14.9 months Median OS—42.7 months
**B. Telomerase (TERT-based)**					
**B.1 Vx-001**					
NCT01935154 [39]	NSCLC expressing hTERT who did not progress after 4 cycles of first-line platinum-based doublet chemotherapy, with HLA-A*0201 haplotype	Vx001 maintenance therapy vs. placebo (peptide vaccine against TERT)	OS	Phase 2 RCT N = 221 (109 in the vaccine arm)	ORR—0.0% DCR—47.2% vs. 43.6% (vaccine vs. placebo) Median OS—14.3 vs. 11.3 months (HR—0.97, 95% CI 0.70–1.34)
**B.2 UCPVax**					
NCT04263051 [47]	Advanced NSCLC, progressed on first-line chemo-immunotherapy	UCPVax with nivolumab compared with chemotherapy (synthetic peptide vaccine against TERT)	6-month PFS	Phase-2 RCT N = 46 32 in vaccine arm, 14 in standard arm	Vaccine vs. standard arm ORR—3.1% vs. 21.0% DCR—15.6% vs. 78.6% Median PFS—1.7 vs. 5.4 months 6-month PFS—9.7% vs. 30.8% Median OS—7.1 vs. 9.7 months Stopped prematurely due to a lack of efficacy
NCT02818426 [52]	Refractory NSCLC progressed on 1–3 lines of chemotherapy and immune checkpoint inhibitors	Universal cancer peptide-based vaccine (UCPVax) (peptide vaccine from highly selected MHC-II binding peptides derived from TERT, called UCP)	DLT, immune response	Phase 1b/2 N = 59	Grade 3+—2.5% ORR—1.9% DCR—38.9% Median PFS—2.2 months Median OS—9.7 months
**B.3. UV1**					
NCT01789099 [40]	Advanced NSCLC without disease progression after at least 1 line of doublet chemotherapy and/or radiotherapy	UV1 synthetic peptide vaccine and GM-CSF (synthetic peptide vaccine against human telomerase reverse transcriptase, hTERT)	Safety and tolerability of UV1 Immunological response	1/2a N = 18	Grade-3 AE—16.6% ORR—5.9% Median PFS—10.7 months Median OS—28.2 months
**B.4 AST-VAC2**					
NCT03371485 [58]	NSCLC (metastatic or locally advanced) with no other suitable treatment options and HLA A*02:01 positive	AST-VAC2 (allogeneic dendritic cell vaccine targeting hTERT)	AEs	1 N = 8 (9 in ITT)	SAE—0.0% ORR—0.0% CBR—62.5% 2-year OS—37.5%
**C. Multi-epitope peptide vaccines—OSE2101**					
NCT02654587 [46]	HLA-A2 positive advanced *EGFR/ALK*-negative NSCLC, progressed on immune checkpoint inhibitors	OSE2101 vs. standard of care chemotherapy (2:1) (multi-epitope peptide vaccine against five tumor-associated antigens—*Her2/neu*, CEA, MAGE 2, MAGE 3, and p53)	OS	Phase 3 N = 219 (139 in vaccine arm)	Vaccine vs. standard-of-care Grade 3+ AE—11.4% vs 35.1% ORR—7.7% vs. 18.4% Median PFS—2.7 vs. 3.0 months (HR 1.28; 95% CI 0.82–2.00; *p* = 0.29) Median OS—8.8 vs. 8.3 months (HR 0.86; 95% CI 0.62–1.19; *p* = 0.36)
**D. Escape/novel antigen targeting**					
NCT05898763 [48]	HLA-A*0201-positive patients with NSCLC who progress after checkpoint blockade	TEIPP24 (with pembrolizumab in the extension cohort) (peptide vaccine against T-cell epitopes associated with impaired peptide processing, LRPAP1)	Safety, tolerability, and immunogenicity	Phase 1/2 N = 26	SAE—34.6% ORR—3.8% CBR—34.6% Median PFS—2.1 months Median OS—9.4 months

AE, adverse event; *ALK*, Anaplastic Lymphoid Kinase; CBR, clinical benefit rate; CI, confidence interval; DCR, disease-control rate; *EGFR*, Epidermal Growth Factor Receptor; HR, hazard ratio; ITT, intention-to-treat; ORR, objective/overall response rate; OS, overall survival; PFS, progression-free survival; SAE, serious adverse event.

**Table 2:** Completed clinical trials on personalized peptide, dendritic cell-based, and whole-tumor-cell vaccines.

NCT Number	Population	Intervention	Primary Outcome	Phase and Sample Size	Results
**A. Personalized peptide vaccines**					
UMIN 000003521 [59]	*EGFR*-negative stage IIIB/IV NSCLC, both treatment naïve and post 1–2 lines of systemic therapy	Docetaxel with Personalized peptide vaccination or placebo (1:1) (personalized peptide vaccine)	PFS	Phase 2 N = 50 (26 in vaccine arm)	Vaccine vs. placebo ORR—3.8% vs. 8.3% DCR—11.5% vs. 20.8% Median PFS—59 vs. 53 days (HR 0.78, 95% CI 0.43–1.42, *p* = 0.42) Median OS—320 vs. 223 days (HR 0.80, 95% CI 0.42–1.51, *p* = 0.49)
NCT03380871 [44]	Advanced treatment-naïve non-squamous NSCLC	NEO PV-01 + carboplatin + pemetrexed + pembrolizumab (personalized neoantigen peptide vaccine)	Adverse events and severe adverse events leading to treatment discontinuation	Phase 1 N = 21 (38 in ITT)	Well tolerated, <5% severe adverse effects, ORR—69.0% (includes effect of chemotherapy and Pembrolizumab) Median PFS—7.2 months Median OS—20.0 months
NCT02897765 [51]	Bladder, advanced melanoma or NSCLC (unresectable, smoking-related NSCLC with 1 one line of systemic chemo in the metastatic setting)	NEO-PV-01 + nivolumab (personalized neoantigen peptide vaccine)	Safety and tolerability	1 N = 18 (NSCLC cohort)	SAE 42.7% (all patients) ORR—39.0% (amongst NSCLC) Median PFS—8.5 months Median OS—not reached (1-year OS 83%)
NCT03633110 [60]	NSCLC completed definitive treatment or is receiving or will receive immunotherapy for stage IV disease	GEN-009 alone for locally advanced or in combination with pembrolizumab or nivolumab (adjuvant personalized cancer vaccine)	TEAEs, T-cell response	Phase 1/2 N = 16	Well tolerated Data on responses and survival—awaited
**B. Dendritic Cell-Based Vaccine**					
NCT02470468 [43]	Advanced NSCLC (stage IV unresectable disease)	DCVAC/LuCa + chemotherapy (A) +/− immune enhancers (B) (Interferon-α and Hydroxychloroquine) vs. Standard of Care chemotherapy (C) (autologous dendritic cell vaccine)	PFS between arms A and C	Phase-2 RCT N = 112 (Arms A 45, B 29, C 38)	Grade-3+ TEAE—0% to vaccine ORR—(A) 45.0% vs. (C) 34.0% Median PFS: A—6.7 vs. C—5.6 months Median OS: A—15.5 vs. C—11.8 months (HR 0.55; 95% CI 0.33–0.93, *p* = 0.0232)
NCT03970746 [45]	HLA-A^∗^02:01 positive NSCLC patients Cohort A—Completed resected stage IIA-IIIA NSCLC Cohort B—Treatment naïve advanced NSCLC with PDL1 >= 50%	PDC∗lung01 with or without anti-PD1 immune checkpoint inhibitors (allogenic antigen-presenting platform with a plasmacytoid dendritic cell line)	Safety	Phase I/II N = 73	Grade 3+ AE—26% In advanced NSCLC cohort, ORR—51% Median PFS—9 months
NCT02956551 [53]	Advanced NSCLC, relapsed after multiline therapy	Neo-DCVac (personalized neoantigen peptide-pulsed autologous dendritic cell vaccine)	Safety and efficacy	Phase I (N = 12)	Grade 3+ AE—0% ORR—25% DCR—75% Median PFS—5.5 months Median OS—7.9 months
**C. Whole-tumor-cell/allogeneic vaccines**					
NCT00676507 [35]	Stage III/IV NSCLC patients who did not progress after platinum-based chemotherapy	Belagenpumatucel-L versus placebo (allogenic whole-tumor-cell vaccine with four NSCLC cell lines)	OS	Phase III N = 532 (270 in vaccine arm)	Vaccine vs. Placebo SAE—N= 49 vs. 32 Median PFS—4.3 vs. 4.0 months (HR — 0.99, *p* = 0.947) Median OS—20.3 vs. 17.8 months (HR—0.94, *p* = 0.594)
NCT02439450 [49,50]	Non-small-cell lung adenocarcinoma or squamous cell carcinoma after at least one prior line of therapy. ICI naïve were cohort A, ICI pretreated were cohort B	Viagenpumatucel-L HS-110 and nivolumab or pembrolizumab +/− pemetrexed (allogenic cellular vaccine)	Safety and tolerability	Phase 1b/2 N = 119	SAE = 15.9% ORR—(A)—21.3%, (B)—10.3% Median PFS (A)—1.8 months Median PFS (B)—2.8 months Median OS (A)—24.6 months Median OS (B)—11.9 months
NCT01433172 [61]	Advanced/metastatic adenocarcinoma lung progressed on at least one line of therapy, with no curative options	GM.CD40L vaccine with or without CCL21 (allogeneic whole-tumor-cell-based vaccine with genetically engineered bystander cells [GM-CSF/CD40L])	For phase 1, Safety and tolerability For phase 2, PFS at 6 months	Phase 1/randomized Phase 2 N = 73 (36 in combination arm)	Vaccine vs. Vaccine with CCL21 Grade 3+ TrAE—0% vs. 0% ORR—0% vs. 0%/DCR—47% vs. 37% 6-month PFS—15.2% vs. 9.4% Median PFS—2.4 vs. 3.4 months (HR 0.87; 95% CI 0.52–1.45, *p* = 0.61) Median OS—9.3 vs. 9.5 months (HR 1.25, 95% CI 0.70–2.25, *p* = 0.44)`

AE, adverse event; CI, confidence interval; DCR, disease-control rate; *EGFR*, Epidermal Growth Factor Receptor; HR, hazard ratio; ORR, objective/overall response rate; OS, overall survival; PFS, progression-free survival; SAE, serious adverse event; TEAEs, treatment-emergent adverse event.

**Table 3:** Completed clinical trials on vector-based, nucleic acid-based, protein/antibody-inducing vaccines.

NCT Number	Population	Intervention	Primary Outcome	Phase and Sample Size	Results
**A. Vector/nucleic acid-based vaccines**					
NCT00480025 [26]	Stage IB, II, and IIIA, MAGE-A3 positive NSCLC after complete surgical resection, with or without adjuvant chemotherapy	recMAGE-A3 with AS15 immunostimulant versus placebo (2:1) (recombinant MAGE-A3 vaccine)	DFS	Phase III RCT N = 2312 (1515 in the vaccine arm)	Vaccine vs. Placebo Grade 3+ AE—16% vs. 16% Median DFS—60.5 vs. 57.9 months (HR 1.02, 95% CI 0.89–1.18, *p* = 0.74)
NCT01383148 [41]	Treatment naïve stage IV *EGFR*-negative NSCLC, with ≥50% MUC1 expression in IHC	Platinum-based doublet chemotherapy with TG4010 or placebo (recombinant viral vector vaccine, against MUC1)	PFS	Phase 2b/3 N = 222 (111 in vaccine arm)	Vaccine vs. Placebo SAE—59% vs. 78% ORR—40% vs. 29% Median PFS—5.9 vs. 5.1 months (HR 0.74, 95% CI 0.55–0.98, *p* = 0.019) Median OS—12.7 vs. 10.6 months (HR 0.78, 95% CI 0.57–1.06, *p* = 0.055)
NCT03353675 [42]	Stage IIIB-IV non-squamous NSCLC or delayed relapse of any stage not amenable to curative intent, with PDL1 < 50%	TG4010, chemotherapy, and Nivolumab (recombinant viral vector vaccine, against MUC1)	ORR	Phase 2 N = 44	SAE—63.6% ORR—32.5% DCR—75.0% Median PFS—5.7 months Median OS—14.9 months
NCT02823990 [62]	Non-squamous NSCLC, stage-IIIB/IV, progressed on 1–3 lines, and *EGFR*/*ALK* negative	TG4010 and nivolumab (recombinant viral vector vaccine, against MUC1)	ORR	Phase 2 N = 13	SAE—7.7% ORR—8.3% DCR—25.0% Median PFS—1.3 months Median OS—7.2 months
NCT03164772 [63]	Metastatic NSCLC	BI 1361849 plus durvalumab (A) +/− tremelimumab (B) (mRNA vaccine with 6 antigens: MUC1, survivin, NY-ESO-1, 5T4, MAGE-C2, MAGE-C1)	TEAEs	Phase 1/2 N = 57 (61 in ITT)	SAE—7.0% ORR (A)—26.3%, (B)—11.1% DCR (A)—63.1%, (B)—40.7% Median PFS (A)—2.0 months, Median PFS (B)—1.8 months
**B. Protein/antibody-inducing vaccines**					
RPCEC00000161 [36]	Stage IIIB/IV NSCLC with at least stable disease after 1st line of platinum-based chemotherapy	CIMAvax-EGF vaccine versus best supportive care, as maintenance (2:1) (protein conjugate vaccine targeting EGF, a tumor-associated antigen)	OS	Phase 3 N = 405 (270 in vaccine arm)	Vaccine vs. best supportive care Grade 3-4 AE—25.7% vs. 19.7% Response rates/PFS—not reported Median OS—10.8 vs. 8.9 months (HR 0.82, 95% CI 0.66–1.03, *p* = 0.100)
NCT01460472 [37]	Stage-IIIB/IV NSCLC with at least stable disease after first-line chemotherapy	Racotumomab-Alum Vaccine versus placebo (1:1) (anti-idiotype vaccine targeting the NeuGcGM3 tumor-associated ganglioside)	OS	Phase 2/3 vs. placebo N = 176 (87 in vaccine arm)	Vaccine vs. placebo SAE— 39.5% vs. 34.8%Median PFS—5.3 vs. 3.9 months (HR 0.73, 95% CI 0.53–0.99; *p* = 0.039) Median OS—8.2 vs. 6.8 months (HR 0.63; 95% CI 0.46–0.87; *p* = 0.004)
RPCEC0000017 [38]	Stage-IIIB/IV NSCLC with at least stable disease after first-line chemotherapy	Racotumomab-Alum Vaccine versus Nimotuzumab vs. docetaxel (2:2:1) (anti-idiotype vaccine targeting the NeuGcGM3 tumor-associated ganglioside)	OS at 1-year (non-inferiority compared to Docetaxel)	Phase 3 N = 232 (93 in vaccine arm)	Vaccine vs. Nimotuzumab vs. Docetaxel SAE—19.4% vs. 25.8% vs. 11.5% DCR at 3 months— 55.8% vs. 50.0% vs. 56.4% Median PFS—4.4 vs. 4.6 vs. 4.0 months (*p* = 0.578 and *p* = 0.203) 1-year OS—43.5% vs. 47.8% vs. 31.0% Median OS—9.8 vs. 11.2 vs. 8.6 months

AE, adverse event; *ALK*, Anaplastic Lymphoid Kinase; CI, confidence interval; DCR, disease-control rate; DFS, disease-free survival; *EGFR*, Epidermal Growth Factor Receptor; HR, hazard ratio; IHC, immunohistochemistry; ITT, intention-to-treat; ORR, objective/overall response rate; OS, overall survival; PDL1, Programmed Death Ligand 1; PFS, progression-free survival; SAE, serious adverse event; TEAEs, treatment-emergent adverse event.

**Table 4:** Therapeutic vaccines in NSCLC: ongoing clinical trials.

NCT Number	Population	Intervention	Primary Outcome	Phase
NCT01720836	Stage I-IIIB NSCLC after surgery or radiotherapy, after adjuvant chemotherapy, or after concurrent chemoradiation	MUC1 100mer peptide vaccine	Immunologic response	1/2
NCT05751798	Stage IV squamous or non-squamous NSCLC (*ALK*, *ROS1*, *EGFR* negative with PDL 1 at least 50%)	Anti-PD-1 OSE-279 +/− peptide vaccine OSE2101	DLT, safety, tolerability	1/2
NCT05242965	Stage IV non-squamous or squamous NSCLC	Plasmid DNA vaccine CD105/Yb-1/SOX2/CDH3/MDM2-polyepitope (STEMVAC)	Change from baseline percentage of CD8+ TIL, AEs	2
NCT05195619	Metastatic, recurrent and/or unresectable NSCLC from stage IIIA (not amenable to radical treatment) to stage IVB	Autologous dendritic cell vaccine loaded with personalized peptides (PEP-DC vaccine) plus cyclophosphamide	No. of patients who received vaccine, AEs, treatment limiting toxicities	1
NCT06015724	Advanced NSCLC with KRAS mutation	Daratumumab plus KRAS vaccine (Targovax TG-01/Stimulon QS-21) plus nivolumab	ORR	2
NCT07285434	IIIB/IIIC/IV or recurrent/metastatic NSCLC	Microlyvaq (Personalized Multi-Epitope Vaccine) plus chemoimmunotherapy	OS, ORR	1
NCT05254184	Stage III/IV unresectable KRAS-mutated NSCLC (Adenocarcinoma)	Pooled Mutant KRAS-Targeted Peptide Vaccine + nivolumab and ipilimumab + chemotherapy	Safety (AEs)	1
NCT03546361	Stage IV NSCLC	Dendritic Cell-Adenovirus CCL21 Vaccine plus pembrolizumab	MTD, maximum administered dose	1
NCT04147078	Localized NSCLC after surgery or ablation	Neoantigen-primed dendritic cell cell vaccine	DFS	1
NCT05104515	Metastatic or locally advanced inoperable NSCLC	OVM-200	Safety and tolerability	1, n = 12
NCT06472245	Stage IV squamous or non-squamous NSCLC	OSE2101	OS	3
NCT05557591	Stage IIIB or stage IIIC disease who are not candidates for definitive treatment or stage IV with no systemic treatment for recurrent or metastatic NSCLC	BNT116 + cemiplimab	ORR	2
NCT05142189	Unresectable Stage III or metastatic Stage IV NSCLC Resectable Stage II and Stage III	mRNA vaccine BNT116 monotherapy or combined with either immunotherapy, chemoimmunotherapy, antibody-drug conjugate, bispecific antibody, or tyrosine kinase inhibitor	DLT, TEAEs, AEs, treatment-related delays	1
NCT02955290	Advanced/unresectable NSCLC	Nivolumab/Pembrolizumab + CIMAvax IM (Recombinant Human EGF-rP64K/Montanide ISA 51 Vaccine) (anti-EGF vaccine)	DLT, OS, PFS	1/2,
NCT02432963	Advanced (unresectable) solid tumors failed or intolerant to one line of treatment	Modified Vaccinia Virus Ankara Vaccine Expressing p53 + Pembrolizumab	AEs	1
NCT06253520	KRAS G12D or G12V genetic mutations-positive stage IV solid tumors	KRAS TCR-Transduced Peripheral blood lymphocyte + GRT-C903/GRT-R904 mRNA vaccine + aldesleukin + fludarabine + cyclophosphamide	Clinical response rate	1
NCT05098210	Unresectable stage III or stage IV NSCLC	Neoantigen Peptide Vaccine + nivolumab + poly ICLC	AEs	1
NCT05344209	Inoperable advanced or metastatic non-small-cell lung cancer	Pembrolizumab or Atezolizumab or cemiplimab + UV1 (peptide-based vaccine)	PFS	2
NCT05269381	Stage II/III non-small-cell lung cancer patients after surgery and other solid tumors	Neoantigen Peptide Vaccine + Pembrolizumab	AEs, MTD, DLT, DFS	1/2
NCT04503278	CLDN6-positive relapsed or refractory advanced solid tumors	CLDN6 CAR-T+/− uRNA-LPX/CLDN6 modRNA-LPX (RNA based vaccine)	TEAEs, DLT, dose reduction or discontinuation	1

AE, adverse event; *ALK*, Anaplastic Lymphoid Kinase; DFS, disease-free survival; DLT, dose-limiting toxicity; *EGFR*, Epidermal Growth Factor Receptor; EFS, event-free survival; *KRAS*, Kirsten Rat Sarcoma Virus; MTD, maximum tolerated dose; ORR, objective/overall response rate; OS, overall survival; PDL1, Programmed Death Ligand 1; PFS, progression-free survival; *ROS1*, c-ros oncogene 1; SAE, serious adverse event; TEAEs, treatment-emergent adverse event.

## 9. Conclusions

Cancer vaccines remain an active area of investigation in NSCLC. While several vaccine platforms, including peptide-, dendritic cell-, nucleic acid-, and whole-cell-based approaches, have demonstrated acceptable safety and immunogenicity, most large randomized studies have not shown a consistent survival benefit. The field has evolved from shared tumor-associated antigens towards personalized neoantigen-based strategies. This may better exploit the advances in genomic profiling and immunotherapy. Ongoing studies combining vaccines with ICIs and other immunomodulatory approaches will help define the future role of vaccines in lung cancer management. At present, therapeutic cancer vaccines remain investigational. However, emerging platforms continue to offer opportunities for improving antitumor immunity and overcoming resistance to immune checkpoint inhibitors.

## Data Availability

No new data were created or analyzed in this study. Data sharing is not applicable to this article.
